# Phylogeographic analysis of the full genome of Sweepovirus to trace virus dispersal and introduction to Korea

**DOI:** 10.1371/journal.pone.0202174

**Published:** 2018-08-13

**Authors:** Jaedeok Kim, Hae-Ryun Kwak, Mikyeong Kim, Jang-Kyun Seo, Jung Wook Yang, Mi-Nam Chung, Eui-Joon Kil, Hong-Soo Choi, Sukchan Lee

**Affiliations:** 1 Crop Protection Division, National Institute of Agricultural Science, Wanju, Korea; 2 Department of Genetic Engineering, Sungkyunkwan University, Suwon, Korea; 3 Graduate school of International Agricultural Technology, Seoul National University, Pyeongchang, Korea; 4 Bioenergy Crop Research Institute, National Institute of Crop Science, Muan, Korea; 5 Research Policy Bureau, Rural Development Administration, Jeonju, Korea; Oklahoma State University, UNITED STATES

## Abstract

Sweet potato is a vegetatively propagated crop that is produced for both growth in Korean fields and for export out of the country. The viruses that are present in introduced sweet potatoes can spread both domestically and to foreign countries. Determining the time and path of virus movement could help curtail its spread and prevent future dispersal of related viruses. Determining the consequences of past virus and sweet potato dispersal could provide insight into the ecological and economic risks associated with other sweet potato-infecting viral invasions. We therefore applied Bayesian phylogeographic inferences and recombination analyses of the available Sweepovirus sequences (including 25 Korean Sweepovirus genomes) and reconstructed a plausible history of Sweepovirus diversification and movement across the globe. The Mediterranean basin and Central America were found to be the launchpad of global Sweepovirus dispersal. Currently, China and Brazil are acting as convergence regions for Sweepoviruses. Recently reported Korean Sweepovirus isolates were introduced from China in a recent phase and the regions around China and Brazil continue to act as centers of Sweepovirus diversity and sites of ongoing Sweepovirus evolution. The evidence indicates that the region is an epidemiological hotspot, which suggests that novel Sweepovirus variants might be found.

## Introduction

According to previous survey results, novel sweet potato infecting virus species were introduced and emerged in Korea between 2003 and 2012 [1,2]. There have been many reports on ‘emerging’ plant viruses, especially *Begomovirus* spp [3–5]. A ‘new’ emerging virus can be easily identified when it is first introduced into a region; however, additional genetic study is required to identify the origin of the emerged virus subpopulation once it has mixed with the older endemic subpopulation, and few reports are available on plant virus species other than *Cucumber mosaic virus* (CMV) [6].

Phylogeographic analysis examines the congruence between phylogenetic and geographic relationships among organisms, thereby elucidating the processes underlying the genetic diversity of populations in space and time. In animal- and human-infecting viruses, phylogenetic analyses provide insight into invasion points and dispersal patterns [7–10]. Likewise, several plant virus species have shown genetic differences among isolates that can be correlated with their geographic origins [6,11,12]. However, the relationship between genetic variation and the geographic origins can be difficult to discern because of virus dispersal via humans and other vectors [13]. Nevertheless, several studies on genetic populations using sequence-based analysis have been reported and many studies on plant viruses have used molecular markers[14,15].

The sweet potato-infecting virus can be used as a molecular marker for sweet potato dispersion. Unlike other virus hosts, sweet potatoes are propagated through vegetative planting material transported between growing fields. Sweet potato is a self-incompatible plant and a highly heterozygous polyploid crop. For this reason, providing seeds of breed-selected sweet potato cultivar lines is not suitable for sweet potato farming. Therefore, planting materials of sweet potato are provided by a tuberous root stock or vegetatively propagated sprouts. In sweet potato producing countries, farmers use vegetatively propagated sprouts as the main materials for further propagation [14]. Roots can be stored for a few months; in this way, foreign sweet potato cultivars can be transported long distances and maintain their growth status of the previous year. Thus, if newly introduced propagation materials are infected with viruses, the viruses will be transmitted with the propagation material to the newly planted field and emerge in new places. Therefore, analysis of novel emerging sweet potato viruses can be used as a remarkable molecular marker of sweet potato introduction compared to other plant viruses.

Over 20 species of viruses are known to infect sweet potatoes, and all are included in the genera *Potyvirus*, *Crinivirus*, *Begomovirus*, and *Cavemovirus* [16]. Among these, sweet potato-infecting *Begomovirus*, named Sweepovirus [17], is a good candidate molecular marker for sweet potatoes. Sweet potato leaf curl disease was first reported in Taiwan and Japan [18], and a similar disease has been observed in Israel [18]. *Sweet potato leaf curl virus* (SPLCV) was the first completely sequenced *Begomovirus* in sweet potato reported in the United States [19,20]. Sweepoviruses have been reported in many countries including Peru, Italy, Spain, China, Korea, Kenya, Uganda, India, and Brazil [1,4,21–29]. Complete genome sequences of SPLCV isolates in Korea have been characterized, and their phylogenetic relationships with other Sweepovirus species have been determined [30,31]. Moreover, complete genome information of Sweepovirus is the most plentiful virus species among all sweet potato-infecting viruses [28,32]. In this study, the spread of Sweepoviruses was analyzed via phylogeographic analysis to estimate the recent origin of this sweet potato-infecting virus in Korea as a molecular marker for introduced sweet potato.

## Materials and methods

### Isolate collection and rolling circle amplification for SPLCV genome sequencing

To determine full-length genome sequences, the following 25 samples were obtained from sweet potato samples collected from 10 regions of five Korean provinces in 2011 and 2012 [2]. Rolling circle amplification (RCA) was performed to amplify the whole Sweepovirus genome using the illustra TempliPhi 100 Amplification Kit (GE Healthcare Co., USA) [33]. The general procedure for RCA reaction of a *Geminivirus* described by Inoue-Nagata *et al*. was followed. The reaction was performed with from 0.5μg to 1μg Sweepovirus-positive sample DNA in 1 μl and was mixed with 5 μl sample buffer and denatured at 95°C for 5 minutes. The denatured reaction mixture was chilled at 4°C for 5 minutes, and then 5 μl reaction buffer and 0.2 μl enzyme mixture were added to the denatured reaction mixture. The final reaction mixture was kept at 30°C for 16 hours to amplify the genome concatamer. The RCA products obtained were treated with the restriction enzyme *Sac* I for 2 hours at 37°C. Restriction enzyme-treated RCA products were visualized on 1% agarose gels containing ethidium bromide. DNA bands of about 2.8 kb—the typical Sweepovirus genome size—were eluted and purified from the agarose gel, and cloned into a *Sac* I-treated pGEM-3zf plasmid vector (Promega Co., USA) with T4 DNA ligase (Promega Co., USA). The ligated plasmid was transformed into *Escherichia coli* (strain DH5α). The cloned constructs were sequenced by a commercial sequencing service (Macrogen Co., Korea). The M13 primer set (5 pM/μl each) and four kinds of primer sets were used for sequencing (S1 Table). The resultant sequences were compared to known sequences using the nucleotide BLAST (BLASTn) algorithm, from “Others” database with “Highly similar sequences (megablast)” in the NCBI database to identify the characterized virus genome.

### Phylogenetic and recombination analyses

Twenty-five new SPLCV and Sweet Potato Golden Vein-associated Virus (SPGVaV) genomes were aligned with all of the full-length Sweepovirus sequences available in GenBank in March 2015 (S2 Table) using the MUSCLE algorithm implemented in GENEIOUS software. Maximum likelihood phylogenetic trees were constructed using RaxML, which was implemented in GENEIOUS software with the model GTR+G4 with 1000 replications of bootstrap iterations.

To identify the parental isolates to verify the origin of virus dispersion, recombination events and breakpoints were predicted with RDP, GENECONV, BOOTSCAN, MAXIMUM CHI SQUARE, CHIMAERA, SISCAN, and 3SEQ recombination detection methods implemented in the RDP4 program. The analysis was carried out with default options for general recombination detection options—the highest acceptable p-value as 0.05 with Bonferroni correction, for permutation options–number of permutations as 0 with using SEQGEN parametric simulations, and for data processing options–with requiring topological evidence, polish breakpoints, with checking alignment consistency and group recombinants realistically options. Only events detected with more than four methods were considered credible evidence of recombination and analyzed in Korean isolates. All the detected recombination events were checked manually and adjusted as the most ancient isolates as the only parents of the all detected recombination events where necessary using the extensive phylogenetic and recombination signal analysis features available in RDP4.

### Phylogeographic analysis and evolutionary rate estimation

The movement patterns of Sweepovirus were reconstructed using the implemented analysis package “GEO_Sphere” for Spherical Phylogeography in BEAST v2.3.2. Phylogeographic analysis was carried out with continuous-time Markov chain models of discrete state evolution to determine the most probable geographic locations of ancestral sequences. Analysis was performed via 200 million steps of the Markov Chain Monte Carlo (MCMC) method to build a posterior tree. The maximum clade credibility tree was annotated with geographical locations using the software TreeAnnotator (available in the BEAST package). While the annotation, TreeAnnotator carried out 10% burn-in of all built trees and summarization to remove the non-significant result trees. After summarization, the resulting tree was visualized using FigTree (ver. 1.3.1). Processed data sets were visualized using the “SPREAD” program on Google Earth to produce a graphical animation of the continuous movement dynamics of Sweepovirus isolates in the key markup language (kml) file format [34]. These kml files, available as S1 File, contain information on the routes and times of virus movements, and can be viewed using Google Earth (available from http://earth.google.com).

## Results

### Phylogenetic relationship of Korean Sweepovirus with isolates worldwide

In 2011 and 2012, virus-infected sweet potato samples were collected and their virus infection status was determined. Among the virus-positive samples, SPLCV-infected samples were chosen for full genome characterization and RCA methods were applied to assure a one-mer genome set. In total, 25 isolates of Sweepovirus genomes were characterized and their phylogenetic relationship with previous Korean and worldwide Sweepovirus isolates was analyzed. *Tomato pseudo-curly top virus* (Genus *Curtovirus*, Geminivirdae) was used as an out-group (accession no. X84735 in GenBank at NCBI).

**Fig 1:**
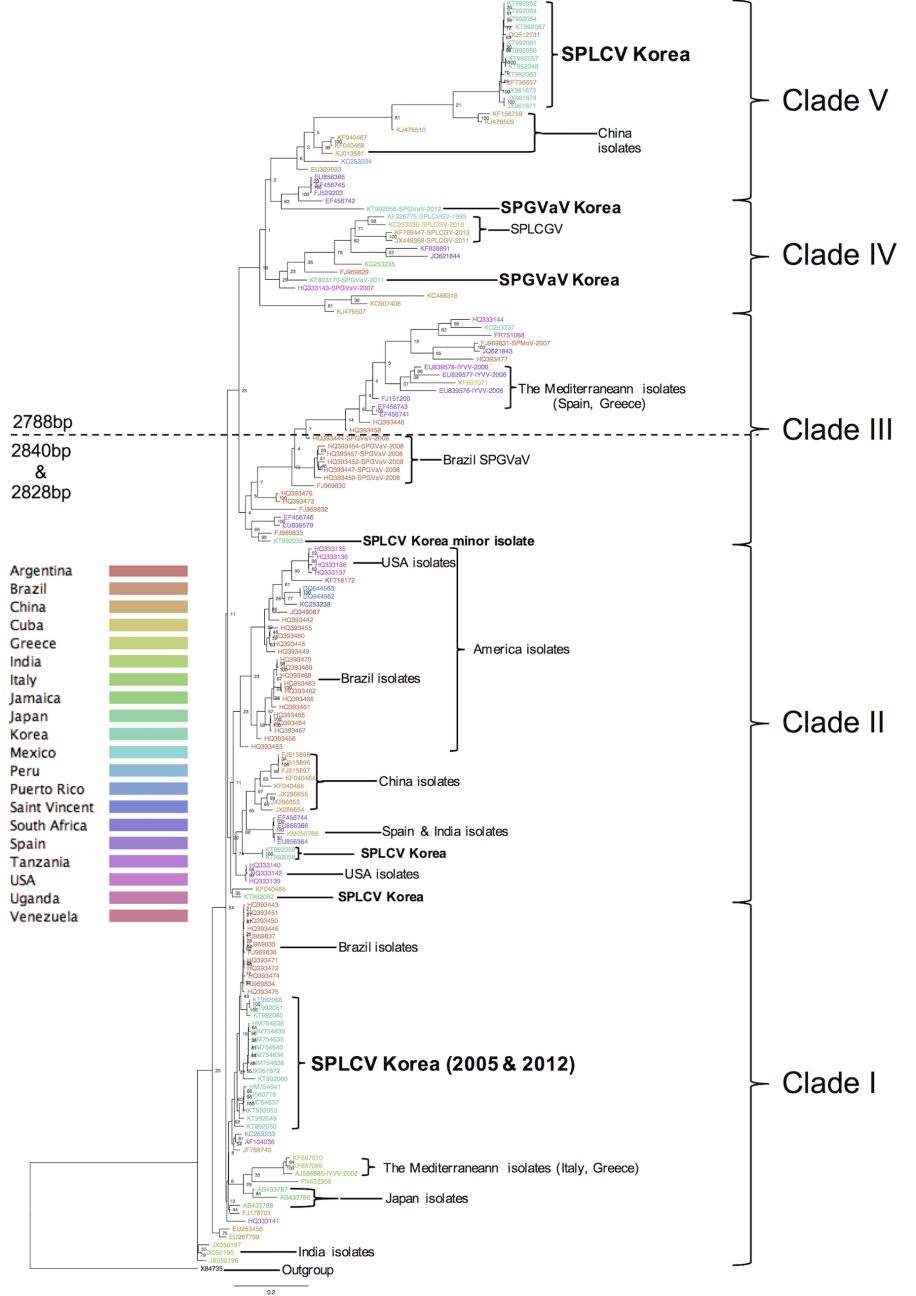
Phylogenetic Sweepovirus tree built with RaxML methods. The tree was divided into 5 clades. Korean isolates spanned all five clades. All Korean isolates are highlighted beside their taxon name on the phylogenetic tree. Taxon names are color coded by country of origin, as indicated to the left of the tree. SPLCV isolates were omitted species name on their taxon name. A crossed line separates the analyzed isolates by genome size. The bootstrap support value is displayed on each branch node. A *Tomato pseudo curly top virus* (Curtovirus) isolate was set as the outgroup.

Sweepovirus isolates were categorized into 5 major clades. Korean isolates spanned all five clades. Clade I included the oldest SPLCV isolates from Japan—AB433786 (1996), AB433787 (1998), and AB433788 (1998)—and the first SPLCV genome characterized in the USA. More than half (17 isolates) of the Korean SPLCV isolates were in this clade and showed a close relationship with AF104036, USA, 1999. All isolates collected in 2005 (FJ560719 and HM754634 to HM754741) were included in this clade. Three isolates collected in 2012, KT992058, KT992051, and KT992065, showed a closer relationship to the Brazilian isolates that were ~2828 bp in size and were collected in 2009. Other 2828-bp-sized Korean isolates collected in 2011 and 2012 were categorized into two sub-categories showing a closer relationship to the USA isolate in 1999, AF104036. The isolates in this clade showed 95 to 100% nucleotide identity with each other. Full genome sequences of Clade I isolates differed by about 10% from Clade II, 15% from Clade III and IV, and 18% from Clade V (Fig 1 and S3 Table).

Isolates from America were included in Clade II and 2828-bp-sized Chinese isolates were also categorized in this clade. Two Korean isolates, KT992059 and KT992062, were included in this clade and showed a close relationship to the Chinese isolates in this clade. Sequence similarity of KT992062 with the Chinese isolate, KF040464, was 94% and that of KT992059 with the Chinese isolate, KF040466, was 93%. Isolates from Brazil, Saint Vincente, Venezuela, and Puerto Rico were separated from the Chinese and Korean isolates in this clade. The US isolates were separated into two groups in this clade according to their genome size. HQ333139, HQ333140, and HQ333142 isolates were 2828 bp in size, whereas isolates HQ333135-38 were about 2795 bp in size. Clade II isolates differed by ~13% from Clade III, ~18% from Clade IV, and ~20% from Clade V (Fig 1 and S3 Table).

Clade III included Spanish *Ipomoea Yellow Vein Virus* (IYVV) and African isolates from Uganda and South Africa. SPGVaV and *Sweet Potato Mosaic-associated Virus* (SPMoV) isolates from Brazil were also included in this clade, along with Spanish SPLCV isolates. One Korean isolate, KT992055, was also categorized in this clade. In contrast to other clades, genome sizes in this clade varied. IYVV and its closely related isolates were 2788 bp or 2800 bp in size. The SPGVaV isolates and SPLCV isolates from Brazil were 2840 bp in size. The 2844-bp Korean isolate in this clade showed a close relationship to the 2840-bp-sized Brazilian Sweepovirus isolates. Clade III isolates differed by ~13% from Clade IV and ~20% from Clade V (Fig 1 and S3 Table).

Most of the *Sweet Potato Leaf Curl Georgia Virus* (SPLCGV) isolates were categorized in Clade IV. Isolates from Mexico, China, Cuba, Jamaica, South Africa, Spain, Tanzania, and the USA were included in this clade. The genome size of virus isolates in this clade was primarily ~2760 to 2800 bp. Korean isolates of SPGVaV were also included in this clade. The first characterized Korean SPGVaV isolate, KF803170, showed a close relationship with the US SPGVaV isolate (HQ333143), with 93% similarity. Another Korean SPGVaV isolate, KT992056, was located between Clades IV and V and showed a close relationship to Clade IV. Nucleotide sequence similarity between Clades IV and V was about 80% (Fig 1 and S3 Table).

Clade V included SPLCV isolates from China and Korea 2788 bp in size. In total, 12 isolates collected in 2011 and 2012 were categorized in this clade. The isolates from this clade showed a sequence difference of ~20% from all other Sweepovirus isolates. However, the virus isolates showed high intra-clade identity with over 99% similarity (Fig 1 and S3 Table).

### Recombination event analysis of Korean isolates

Korean Sweepovirus isolates were categorized into five different clades. To compare and analyze the relationship of Korean Sweepovirus isolates to all reported Sweepovirus genomes available in the GenBank database of NCBI, all detected recombination events were re-checked with the oldest Sweepovirus isolates used as the parents of the recombination events. AB433786 (1996), AB433787 (1996), and AB433788 (1998) from Japan, AF104036 (1999) from the USA, and AF326775 (1999) from Mexico were designated as the parents of all detected recombination events. In total, eight types of recombination events were detected in 18 Korean isolates (Table 1).

**Table 1:** Recombination events in complete Korean Sweepovirus genomes obtained using recombination detection software.

Type	Recombinant isolate^a^	Recombination site in genome		Parental isolates^b^	RDP4^c^	*p*-value^d^
		Start	End			
I	KT992051	nt 1763	nt 2748	KT992062 (Korea, 2012) x KT992060 (Korea, 2012)	R**G**MCSL3	1.225 x 10^−07^
		nt 10	nt 411	HQ393475 (Brazil, 2009) x HM754639 (Korea, 2005)	**G**MCSL3	1.275 x 10^−03^
II	KT992065	nt 1960	nt 2748	KT992062 (Korea, 2012) x KT992060 (Korea, 2012)	R**G**MCSL3	1.225 x 10^−07^
		nt 2749	nt 862	HQ393475 (Brazil, 2009) x HM754639 (Korea, 2005)	**G**MCSL3	1.275 x 10^−03^
III	KT992068	nt 16	nt 734	HM754639 (Korea, 2005) x HQ393475 (Brazil, 2009)	**G**MCSL3	1.275 x 10^−03^
		nt 1960	nt 2748	KT992060 (Korea, 2012) x KT992062 (Korea, 2012)	RGMCSL**3**	1.592 x 10^−04^
IV	KT992062	nt 2181	nt 2810	KT992050 (Korea, 2012) x HQ333142 (Brazil, 2009)	GMSL**3**	1.208 x 10^−03^
V	KT992059	nt 1325	nt 1800	JX050197 (India, 2009) x JX286654 (China, 2012)	G**M**L3	6.552 x 10^−01^
VI	KF803170	nt 1922	nt 2809	KT992053 (Korea, 2012) x JX448368 (China, 2011)	**R**GBMCSL3	1.559 x 10^−10^
VII	KT992056	nt 2283	nt 2746	KF040466 (China, 2012) x HQ333142 (Brazil, 2009)	GB**M**CSL3	1.467 x 10^−13^
		nt 2096	nt 2824	KT992053 (Korea, 2012) x JX448368 (China, 2011)	**R**GBMCSL3	1.559 x 10^−10^
VII	JX961671 JX961673 JX961674 KT992048 KT992052 KT992054 KT992061 KT992063 KT992064 KT992066	nt 1060	nt 2046	KF836891 (Tanzania, 2012) x FN432356 (India, 2008)	**G**MCL3	1.947 x 10^−5^

^a^Some recombinants seemed to be ‘tentative’ or originated from a common ancestral virus; e, ‘tentative’ because of support from fewer than three methods; f, ‘tentative’ because one of the parental isolates is ‘unknown’.

^b^‘Parental isolates’ indicates the most likely isolate among those analyzed.

^c^RDP4-implemented methods supporting the corresponding recombination sites; R (RDP), G (GENECONV), B (BootScan), M (MaxChi), C (Chimaera), S (SiScan), P (PhylPro), L (LARD), and 3 (3Seq).

^d^The reported *p*-value is the highest among those calculated using RDP4-implemented methods; the corresponding method is shown in bold.

Among the 17 Korean isolates in Clade I, only three isolates were recombinants. No recombination events were detected in any previous Korean SPLCV isolates, the five SPLCV isolates collected in 2011 and 2012, the Japanese isolates, and the oldest SPLCV isolates from the USA. Among other Korean isolates in Clade I, three isolates, KT992051, KT992065, and KT992068, were predicted to be recombinants of Korean isolates and of the Korean and Brazilian isolates in Clade I (Fig 1 and Table 1). Recombination types I, II, and III were predicted recombination events in the Korean SPLCV isolates from Clade I. Each type of recombinant had two kinds of recombination events predicted within the same parental isolates, whereas the predicted break points were not identical. However, the Brazil isolate, HQ393475, which was predicted to be the parental recombination event, was identical to Korean isolates with 97% similarity (S3 Table). Therefore, all three Korean isolates might be recombinants of the former Korean SPLCV isolates.

Two isolates in Clade II were detected with different recombination types. A type IV event was predicted with KT992062 as a recombinant between the Korean SPLCV isolates and the Brazilian isolate, HQ33142, which showed high similarity of 97% with the Korean SPLCV isolates. Similar to the recombinants in Clade I, KT992062 seemed to be a recombinant of the Korean SPLCV isolates. A type V recombination event was predicted with the KT992059 isolate as a recombinant between the Indian isolate, JX050197, and the Chinese isolate, JX286654 (Fig 1 and Table 1).

SPGVaV Korean isolates were also found to be recombinants. A type VI event in KF803170 was predicted to represent recombination between Korean SPLCV isolates and the Chinese SPLCGV isolate, JX448368, and type VII events were indicated in the KT992056 isolate with two kinds of recombination events predicted in one isolate. The first event was recombination between the Chinese SPLCV isolate, KF040466, and the USA isolate, HQ333142, and the other recombination event was between the Korean isolates and the Chinese isolate, which was at a location similar to the type VI recombination event.

A type VIII event was revealed for all Korean SPLCV isolates in Clade V. This type of event represented recombination between the KF836891 (Tanzania, 2012) and FN432356 (India, 2008) isolates. This event was also observed in the Chinese isolates DQ512731 and JF736657, with the exact predicted location and recombination parents with high similarity of 99%. Therefore, this type of recombination event might be strong evidence for migration of the virus and its carrying host from China to Korea.

In summary, when previous Korean SPLCV isolates were excluded, all newly characterized Sweepovirus isolates in Korea showed a close relationship to Chinese Sweepovirus isolates regardless of their phylogenetic relationship.

### Phylogeographic analysis of the full-length Sweepovirus genome to trace virus dispersal

**Fig 2:**
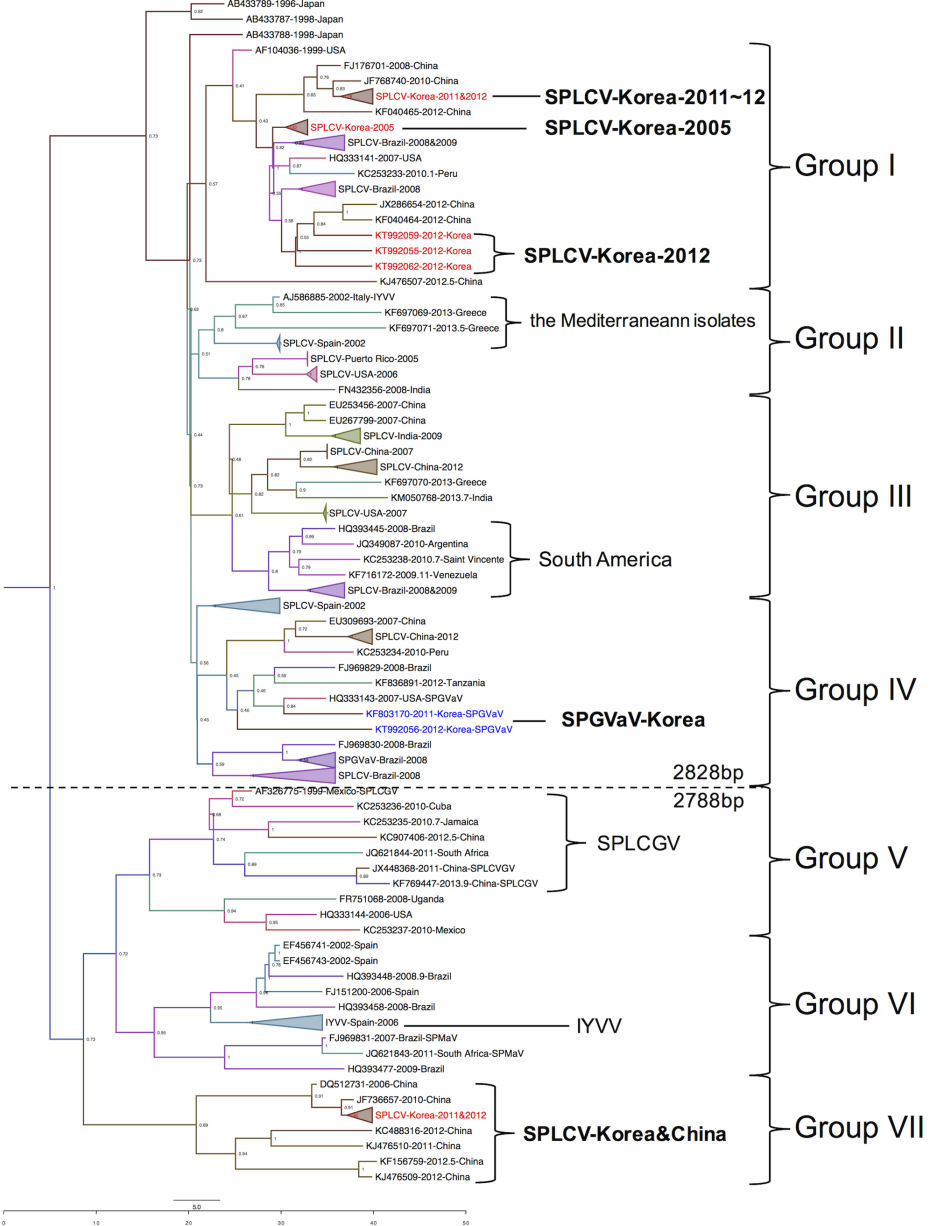
The maximum clade credibility tree for Sweepovirus. All analyzed isolates were categorized into two major lineages with seven groups. Separation of the two lineages was supported by genome size correlated with the analyzed virus species. Isolates located on the same branch with the same countries of origin were collapsed and labeled with the summarized taxon name. The X-axis represents the year of collection.

To trace virus dispersal patterns and the route of recent introductions to Korea, phylogeographic analysis was performed with full-genome sequences adjusted for their collection region and date information obtained from GenBank in the NCBI database. According to the MCC tree results, Sweepoviruses were categorized into seven groups. Group I to IV and Group V to VII were separated from each other as two different lineages. Most of the SPLCV and all of the SPGVaV species were categorized into Group I to IV. IYVV and SPLVGV species, as well as Chinese and Korean SPLCV isolates, were included in Group V to VII (Fig 2).

Considering the Korean isolates, phylogeographically analyzed isolates were grouped together within three groups. One of the Korean Sweepovirus groups included Korean SPLCV isolates with a genome size of 2828 bp or 2829 bp and these were included in Group I. In this group, the Korean SPLCV isolates were separated into three sub-groups. Former Korean SPLCV isolates collected in 2005 were categorized together and were distinct from any recent Korean SPLCV isolates. Other Korean SPLCV isolates with a genome size of 2828 bp had a close relationship to Chinese isolates FJ176701 and JF768740. The other three isolates branched away from former Korean SPLCV isolates KT992059, KT992055, and KT992062 and were located in Group I of the MCC tree with a close relationship to Chinese isolates JX286654 and KF040464. KT992059 and KT992062 were also at a distinct branch location corresponding to Group I of the RaxML phylogenetic tree, and the KT992055 isolate had a distinguishing branch location corresponding to Group III of the RaxML phylogenetic tree. All recent Korean SPLCV isolates in Group I showed a close relationship to the Chinese SPLCV isolates.

In Group IV, the Korean SPGVaV isolates branched from the Spanish SPLCV isolate, showing a close relationship to SPGVaV isolates from the USA. Unlike Brazilian isolates of SPGVaV, Korean SPGVaV isolates were located close to Chinese SPLCV isolates. The phylogenetic relationship of this group was similar to that of Clade IV of the RaxML tree, with the similar isolates and their phylogenetic distance (Figs 1 and 2).

In Group VII, Korean isolates were categorized with Chinese isolate DQ512731. All Korean SPLCV isolates in this clade showed high identity to the ancient Chinese isolate, with genome size and sequence similarity of 99%. In the RaxML tree, this clade was significantly separated from any other Sweepoviruses in Clade V.

**Fig 3:**
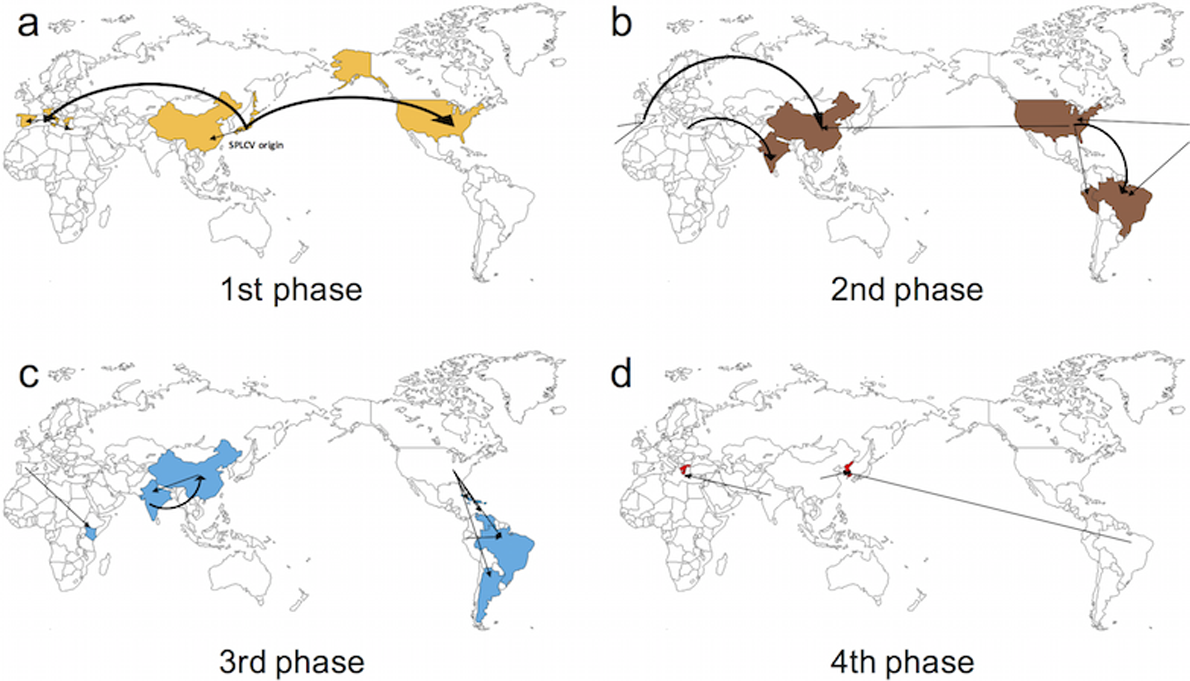
Schematic presentation of continuous geographic SPREAD of Group I to IV on the MCC tree. Arrows represent the predicted migration route of analyzed virus isolates. Estimated migration phases are color coded according to their predicted migration time. a) The first migration phase of Sweepoviruses of Group I to IV. From the most ancient isolates of SPLCV, isolates spread to Italy, Greece, and Spain in the Mediterranean region and to the USA in this phase. b) The second migration phase of Sweepovirus. From the USA, SPLCV migrated to China in Asia and Peru and Brazil in South America. From Spain, the viruses migrated to the USA and Brazil in the Americas. From Greece, the viruses migrated to India in Central Asia. c) The third migration phase of Sweepovirus. Viruses migrated to China and India in Asia and to Brazil and Argentina in South America. The Asian isolates moved to countries between China and India in this phase. d) The fourth migration phase of Sweepovirus. Viruses migrated to Greece and Korea. In this phase, viruses from India migrated to Greece and those from China and Brazil migrated to Korea.

**Fig 4:**
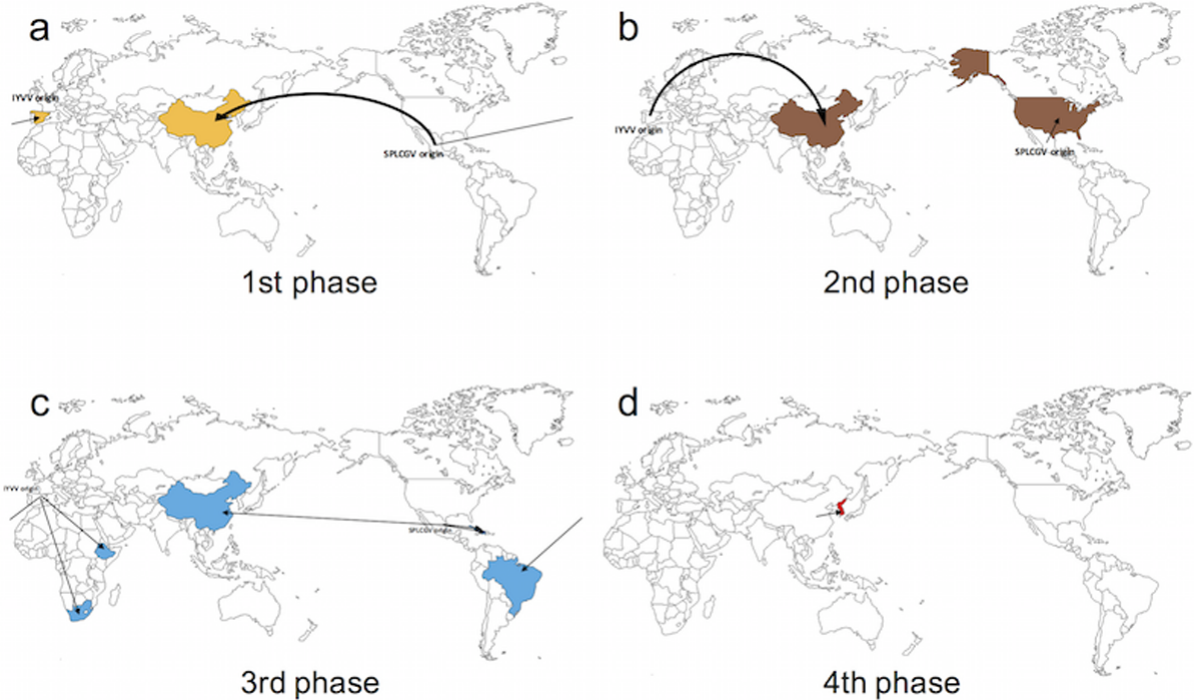
Schematic presentation of the continuous geographical SPREAD of Group V to VII on the MCC tree. Arrows represent the predicted migration pathway of analyzed virus isolates. Estimated times are color coded according to their predicted migration year. a) The first migration phase of Sweepoviruses of Group V to VII. SPLCGV isolates spread from Mexico to Spain and China in this phase. b) The second migration phase. SPLCGV isolates migrated to the USA. IYVV were raised from Spain and migrated to China. c) The third migration phase. Viruses migrated to Africa and South America from Spain. From Mexico, viruses migrated to China and the islands of Central America. d) The fourth migration phase of Sweepovirus. Viruses migrated from China to Korea.

With the annotated MCC tree generated using TreeAnnotator of the BEAST package, continuous geographic spread patterns were visualized on a sphere map of the earth using Google Earth (Google Co.). Continuous spread patterns were drawn on blank maps for two lineages (Figs 3 and 4). In both lineages, the origins of the virus diffusion point were analyzed, based on three countries, the USA, Spain, and China. One of the most ancient isolates was a Japanese isolate in both tree analysis results, the MCC tree and RaxML tree. From the most ancient isolates, the virus seemed to spread out to the three countries mentioned above. The Mediterranean region was the diffusion origin to America, Africa, and central Asia. The USA was a virus convergence region and the origin of spread to other American countries. China was the origin of virus spread to Asian countries. The virus seemed to converge in China in six different ways rather than spreading out to nearby countries. The results showed that SPLCV isolates were introduced to Korea several times after 2006. Three different virus genomes were identified and all showed high similarity to the Chinese isolates. Moreover, Korean SPGVaV isolates had a close relationship in the MCC tree results. Therefore, the newly characterized Sweepovirus isolates from Korea are presumed to have been introduced from China via newly imported sweet potato cultivars.

## Discussion

In our previous survey, sweet potato virus species that had emerged in the past decade were found to be introduced to Korea [1,2,35]. To understand the pathology of the new virus species and strains, their genomes were characterized and compared with the formerly reported virus genome [36]. However, the genome information for the sweet potato-infecting RNA virus was insufficient to trace phylogenetic origins [36]. In contrast, full-genome information for the sweet potato-infecting Begomovirus, Sweepovirus, was plentiful and could be used to compare origins and collection dates [32]. Moreover, sweet potato is not supplied as a seed for planting purposes. Sweet potatoes are propagated in a vegetative manner and thus any virus already present in the propagules can travel with the host as the planting materials are transported. Therefore, we compared the genome sequences of 159 global Sweepovirus isolates in order to assess the spatial genetic structure of the population using different evolutionary assumptions.

Studies of virus properties have generally focused on analyzing phylogenetic relationships and recombination events with the characterized gene or genome sequences [37]. Comparison of the genetic structure of the populations of co-distributed species can provide insight into the extent to which extrinsic and intrinsic factors interact to affect the geographic scale of population differentiation [6,13]. Several studies revealed that the mapping of recombination events as well as phylogenetic relationships can be useful for tracing migration and evolution [6]. On RaxML phylogenetic analysis, newly characterized Korean SPLCV isolates showed a close relationship to Chinese isolates in each of the different clades (Fig 1). All of these categorizations were supported by recombination event predictions. All eight events were correlated with Chinese Sweepovirus isolates as the recombinational parent or as the same recombinants (Table 1). This acted as supporting evidence for the recent migration of viruses from China to Korea. Previous analysis of the sweet potato-infecting *Potyvirus* genome collected from Korea in 2012 also provided similar evidence, pointing to China as the virus introduction origin [36].

Phylogeographic analysis looks for congruence between the phylogenetic and geographic relationships of organisms to elucidate the processes underlying the genetic diversity of populations in space and time. The epidemiology of animal and human viruses has been analyzed using Bayesian algorithm-based methods [7–10,38]. Studies on several plant virus species have also shown that genetic differences correlate with their geographic origins [6,11,13,39]. Bayesian algorithm-based methods have also been applied to investigate dispersal and epidemic patterns [40,41]. In our study, four Sweepovirus lineages originated from three regions regardless of their lineage, and recently characterized Sweepovirus species in Korea were found to be introduced from China (Figs 2, 3, and 4). Although valuable information was obtained from our phylogeographic study, these data should be interpreted in light of their limitations. In the case of the human *Japanese Encephalitis Virus*, virus sample collection was not precise [38]. Sweet potato leaf curling disease was first reported in Taiwan in 1975 [16], while the oldest isolates were collected in Japan in 1996, and the first characterization of the SPLCV genome was reported in 1999 [20]. The RaxML tree (Fig 1) results do not consider the spatial and temporal genome information do support the MCC tree result. Moreover, recent full-length genome analysis results of sweet potato infecting potyvirus revealed a close relationship to Chinese isolates and species [36]. Therefore, phylogeographic analysis results appeared to be reliable for estimating the recent origins of virus introduction, and China was identified as the recent launch-pad of emerging of sweet potato-infecting viruses in Korea.

Because sweet potato cultivars are transported and planted as vegetatively produced vines or tuberous roots, virus already present in these materials could act as a molecular marker for the host plant. Several studies on genetic populations using sequence-based analysis have been reported and studies of plant viruses have used molecular markers [13]. The host properties and sweet potato-infecting virus could be more informative molecular markers than other plant viruses. Roullier *et al* [42] described the diffusion of sweet potatoes in Oceania via phylogenetic analysis of sweet potato chloroplast DNA sequence variation. They determined three possible diffusion routes. The first route was from Central America to the Philippine region rather than to all of Asia. The second route was from Central America to the New Zealand region through the Polynesian islands, and the last route was from Central America, passing through Europe to Africa and then to Asia. Our phylogeographic analysis results showed highly similar patterns between virus spread and sweet potato diffusion (Figs 3 and 4).

In conclusion, our study demonstrated the dispersal of sweet potato-infecting viruses and their host worldwide, with a particular emphasis on Korea. Phylogeographic analyses showed reliable distribution patterns of virus that are strongly supported by phylogenetic and recombination event analyses. Our study suggests that these viruses could be useful molecular markers for introduced sweet potato.

## Supporting information

S1 Table Primers used for Sweepovirus genome sequencing. (DOCX)

S2 Table List of full Sweepovirus genome sequences in this study. (DOCX)

S3 Table Sweepovirus sequence homology matrix (%). (CSV)

S1 File SPREAD pattern of Sweepoviruses. (KML)
