# Supplementary material for: Phylogeographic analysis of the full genome of Sweepovirus to trace virus dispersal and introduction to Korea
Source: PLoS One. 2018 Aug 13;13(8):e0202174. doi: 10.1371/journal.pone.0202174 (PMC6089449; doi:10.1371/journal.pone.0202174)
Supplement: S1 Table — (DOCX) [file pone.0202174.s001.docx]

**S1 Table.** Primers used for Sweepovirus full genome sequencing

| Primer name | sequence(5′🡪3′) | loci |
| --- | --- | --- |
| SPLCV-1F | GAAGAAATACGAGCCAGGAACC | 227-249 |
| SPLCV-1R | TATTTTGGAACGCCTTAAATGGCG | 1249-1226 |
| SPLCV-2F | GTGTATCAGACCCTGCGTTGCA | 1012-1033 |
| SPLCV-2R | CAGTATGGGCCAGGTCTTTGGG | 2007-1985 |
| SPLCV-3F | AGTCCTTCTGGGCCCCCAT | 1848-1867 |
| SPLCV-3R | ACCCAGAGTTGTCGGAATTTGAAT | 2815-2792 |
| SPLCV-4F | AGGTTCCCCATCCTCGTGC | 2524-2542 |
| SPLCV-4R | GGACATAGCTTCGGGCAGC | 436-418 |
