# Supplementary material for: Phylogeographic analysis of the full genome of Sweepovirus to trace virus dispersal and introduction to Korea
Source: PLoS One. 2018 Aug 13;13(8):e0202174. doi: 10.1371/journal.pone.0202174 (PMC6089449; doi:10.1371/journal.pone.0202174)
Supplement: S2 Table — (DOCX) [file pone.0202174.s002.docx]

**S2 Table**. List of the Sweepovirus full genome sequences in this study

| NCBI  accession no. | Origin | Collected Date | Species | Genome size (bp) |
| --- | --- | --- | --- | --- |
| AB433786 | Japan | 1996 | SPLCV | 2844 |
| AB433787 | Japan | 1998 | SPLCV | 2829 |
| AB433788 | Japan | 1998 | SPLCV | 2828 |
| AF104036 | USA | 1999 | SPLCV | 2828 |
| AF326775 | Mexico | 1999 | **SPLCGV** | 2773 |
| AJ586885 | Italy | 2002 | **IYVV** | 2830 |
| DQ512731 | China | 2006 | SPLCV | 2771 |
| DQ644562 | Puerto Rico | 2005 | SPLCV | 2828 |
| DQ644563 | Puerto Rico | 2005 | SPLCV | 2828 |
| EF456741 | Spain | 2002 | SPLCV | 2780 |
| EF456742 | Spain | 2002 | SPLCV | 2806 |
| EF456743 | Spain | 2002 | SPLCV | 2779 |
| EF456744 | Spain | 2002 | SPLCV | 2829 |
| EF456745 | Spain | 2002 | SPLCV | 2807 |
| EF456746 | Spain | 2002 | SPLCV | 2814 |
| EU253456 | China | 2007 | SPLCV | 2800 |
| EU267799 | China | 2007 | SPLCV | 2799 |
| EU309693 | China | 2007 | SPLCV | 2835 |
| EU839576 | Spain | 2006.6 | **IYVV** | 2791 |
| EU839577 | Spain | 2006.6 | **IYVV** | 2783 |
| EU839578 | Spain | 2006.6 | **IYVV** | 2783 |
| EU839579 | Spain | 2002 | SPLCV | 2814 |
| EU856364 | Spain | 2002 | SPLCV | 2829 |
| EU856365 | Spain | 2002 | SPLCV | 2807 |
| EU856366 | Spain | 2002 | SPLCV | 2828 |
| FJ151200 | Spain | 2006.6 | SPLCV | 2781 |
| FJ176701 | China | 2008.6 | SPLCV | 2828 |
| FJ515896 | China | 2007.12 | SPLCV | 2828 |
| FJ515897 | China | 2007.12 | SPLCV | 2828 |
| FJ515898 | China | 2007.12 | SPLCV | 2828 |
| FJ529203 | Spain | 2002 | SPLCV | 2807 |
| FJ560719 | Korea | 2005 | SPLCV | 2828 |
| FJ969829 | Brazil | 2008 | SPGVaV | 2831 |
| FJ969830 | Brazil | 2008 | **SPGVaV** | 2834 |
| FJ969831 | Brazil | 2007 | **SPMoV** | 2803 |
| FJ969832 | Brazil | 2008 | SPLCV | 2841 |
| FJ969833 | Brazil | 2008 | SPLCV | 2845 |
| FJ969834 | Brazil | 2008 | SPLCV | 2829 |
| FJ969835 | Brazil | 2008 | SPLCV | 2841 |
| FJ969836 | Brazil | 2008 | SPLCV | 2829 |
| FJ969837 | Brazil | 2008 | SPLCV | 2829 |
| FN432356 | India | 2008 | SPLCV | 2823 |
| FR751068 | Uganda | 2008.11 | SPLCV | 2799 |
| HM754634 | Korea | 2005 | SPLCV | 2829 |
| HM754635 | Korea | 2005 | SPLCV | 2829 |
| HM754636 | Korea | 2005 | SPLCV | 2829 |
| HM754637 | Korea | 2005 | SPLCV | 2828 |
| HM754638 | Korea | 2005 | SPLCV | 2829 |
| HM754639 | Korea | 2005 | SPLCV | 2829 |
| HM754640 | Korea | 2005 | SPLCV | 2829 |
| HM754641 | Korea | 2005 | SPLCV | 2828 |
| HQ333135 | USA | 2006 | SPLCV | 2795 |
| HQ333136 | USA | 2006 | SPLCV | 2797 |
| HQ333137 | USA | 2006 | SPLCV | 2797 |
| HQ333138 | USA | 2006 | SPLCV | 2975 |
| HQ333139 | USA | 2007 | SPLCV | 2827 |
| HQ333140 | USA | 2007 | SPLCV | 2827 |
| HQ333141 | USA | 2007 | SPLCV | 2829 |
| HQ333142 | USA | 2007 | SPLCV | 2828 |
| HQ333143 | USA | 2007 | **SPGVaV** | 2824 |
| HQ333144 | USA | 2006 | SPLCV | 2782 |
| HQ393442 | Brazil | 2008 | SPLCV | 2827 |
| HQ393443 | Brazil | 2008 | SPLCV | 2829 |
| HQ393444 | Brazil | 2008 | **SPGVaV** | 2834 |
| HQ393445 | Brazil | 2008 | SPLCV | 2828 |
| HQ393446 | Brazil | 2008 | SPLCV | 2829 |
| HQ393447 | Brazil | 2008 | **SPGVaV** | 2834 |
| HQ393448 | Brazil | 2008.9 | SPLCV | 2779 |
| HQ393449 | Brazil | 2008 | SPLCV | 2828 |
| HQ393450 | Brazil | 2008 | SPLCV | 2829 |
| HQ393451 | Brazil | 2008 | SPLCV | 2829 |
| HQ393452 | Brazil | 2008 | **SPGVaV** | 2835 |
| HQ393453 | Brazil | 2008 | SPLCV | 2829 |
| HQ393454 | Brazil | 2008 | **SPGVaV** | 2834 |
| HQ393455 | Brazil | 2008 | SPLCV | 2828 |
| HQ393456 | Brazil | 2008 | SPLCV | 2829 |
| HQ393457 | Brazil | 2008 | **SPGVaV** | 2843 |
| HQ393458 | Brazil | 2008 | SPLCV | 2818 |
| HQ393459 | Brazil | 2008 | **SPGVaV** | 2834 |
| HQ393460 | Brazil | 2008 | SPLCV | 2828 |
| HQ393461 | Brazil | 2009 | SPLCV | 2829 |
| HQ393462 | Brazil | 2009 | SPLCV | 2828 |
| HQ393463 | Brazil | 2009 | SPLCV | 2828 |
| HQ393464 | Brazil | 2009 | SPLCV | 2829 |
| HQ393465 | Brazil | 2009 | SPLCV | 2829 |
| HQ393466 | Brazil | 2009 | SPLCV | 2828 |
| HQ393467 | Brazil | 2009 | SPLCV | 2828 |
| HQ393468 | Brazil | 2009 | SPLCV | 2827 |
| HQ393469 | Brazil | 2009 | SPLCV | 2827 |
| HQ393470 | Brazil | 2009 | SPLCV | 2827 |
| HQ393471 | Brazil | 2009 | SPLCV | 2829 |
| HQ393472 | Brazil | 2009 | SPLCV | 2829 |
| HQ393473 | Brazil | 2009 | SPLCV | 2837 |
| HQ393474 | Brazil | 2009 | SPLCV | 2829 |
| HQ393475 | Brazil | 2009 | SPLCV | 2827 |
| HQ393476 | Brazil | 2009 | SPLCV | 2837 |
| HQ393477 | Brazil | 2009 | SPLCV | 2782 |
| JF736657 | China | 2010 | SPLCV | 2790 |
| JF768740 | China | 2010.8 | SPLCV | 2827 |
| JQ349087 | Argentina | 2010 | SPLCV | 2828 |
| JQ621843 | South Africa | 2011 | **SPMoV** | 2783 |
| JQ621844 | South Africa | 2011 | SPLCV | 2769 |
| JX050195 | India | 2009.8 | SPLCV | 2801 |
| JX050196 | India | 2010.7 | SPLCV | 2801 |
| JX050197 | India | 2009.8 | SPLCV | 2801 |
| JX286653 | China | 2012.5 | SPLCV | 2829 |
| JX286654 | China | 2012.5 | SPLCV | 2829 |
| JX286655 | China | 2012.5 | SPLCV | 2828 |
| JX448368 | China | 2011.7 | **SPLCGV** | 2785 |
| JX961671 | Korea | 2011 | SPLCV | 2788 |
| JX961672 | Korea | 2011 | SPLCV | 2828 |
| JX961673 | Korea | 2011 | SPLCV | 2787 |
| JX961674 | Korea | 2011 | SPLCV | 2788 |
| KC253233 | Peru | 2010.1 | SPLCV | 2829 |
| KC253234 | Peru | 2010 | SPLCV | 2828 |
| KC253235 | Jamaica | 2010.7 | SPLCV | 2767 |
| KC253236 | Cuba | 2010 | **SPLCGV** | 2769 |
| KC253237 | Mexico | 2010.7 | SPLCV | 2784 |
| KC253238 | Saint Vincente | 2010.7 | SPLCV | 2828 |
| KC488316 | China | 2012 | SPLCV | 2764 |
| KC907406 | China | 2012.5 | SPLCV | 2766 |
| KF040464 | China | 2012 | SPLCV | 2836 |
| KF040465 | China | 2012 | SPLCV | 2831 |
| KF040466 | China | 2012.5 | SPLCV | 2826 |
| KF040467 | China | 2012 | SPLCV | 2834 |
| KF040468 | China | 2012 | SPLCV | 2834 |
| KF156759 | China | 2012.5 | SPLCV | 2786 |
| KF697069 | Greece | 2013 | SPLCV | 2830 |
| KF697070 | Greece | 2013 | SPLCV | 2830 |
| KF697071 | Greece | 2013.5 | SPLCV | 2804 |
| KF716172 | Venezuela | 2009.11 | SPLCV | 2829 |
| KF769447 | China | 2013.9 | **SPLCGV** | 2786 |
| KF803170 | Korea | 2011 | **SPGVaV** | 2824 |
| KF836891 | Tanzania | 2012 | SPLCV | 2768 |
| KJ013581 | China | 2012 | SPLCV | 2834 |
| KJ476507 | China | 2012.5 | SPLCV | 2785 |
| KJ476509 | China | 2012 | SPLCV | 2826 |
| KJ476510 | China | 2011 | SPLCV | 2831 |
| KM050768 | India | 2013.7 | SPLCV | 2830 |
| KT992048 | Korea | 2012 | SPLCV | 2787 |
| KT992049 | Korea | 2012 | SPLCV | 2829 |
| KT992050 | Korea | 2012 | SPLCV | 2830 |
| KT992051 | Korea | 2012 | SPLCV | 2828 |
| KT992052 | Korea | 2012 | SPLCV | 2787 |
| KT992053 | Korea | 2012 | SPLCV | 2829 |
| KT992054 | Korea | 2012 | SPLCV | 2787 |
| KT992055 | Korea | 2012 | SPLCV | 2844 |
| KT992056 | Korea | 2012 | **SPGVaV** | 2807 |
| KT992057 | Korea | 2012 | SPLCV | 2787 |
| KT992059 | Korea | 2012 | SPLCV | 2828 |
| KT992060 | Korea | 2012 | SPLCV | 2829 |
| KT992061 | Korea | 2012 | SPLCV | 2786 |
| KT992062 | Korea | 2012 | SPLCV | 2828 |
| KT992063 | Korea | 2012 | SPLCV | 2787 |
| KT992064 | Korea | 2012 | SPLCV | 2787 |
| KT992065 | Korea | 2012 | SPLCV | 2828 |
| KT992066 | Korea | 2012 | SPLCV | 2786 |
| KT992067 | Korea | 2012 | SPLCV | 2787 |
| KT992068 | Korea | 2012 | SPLCV | 2828 |
